# Experiences of and responses to disrespectful maternity care and abuse during childbirth; a qualitative study with women and men in Morogoro Region, Tanzania

**DOI:** 10.1186/1471-2393-14-268

**Published:** 2014-08-12

**Authors:** Shannon A McMahon, Asha S George, Joy J Chebet, Idda H Mosha, Rose NM Mpembeni, Peter J Winch

**Affiliations:** Department of International Health, Johns Hopkins Bloomberg School of Public Health, 615 North Wolfe Street, Baltimore, MD 21205 USA; Behavioural Sciences Department, Muhimbili University of Health and Allied Sciences, PO Box 65015, Dar es Salaam, Tanzania; Department of Epidemiology and Biostatistics, Muhimbili University of Health and Allied Sciences, PO Box 65015, Dar es Salaam, Tanzania

**Keywords:** Maternal health, Abuse, Respectful maternity care, Tanzania, Male involvement, Childbirth

## Abstract

**Background:**

Interventions to reduce maternal mortality have focused on delivery in facilities, yet in many low-resource settings rates of facility-based birth have remained persistently low. In Tanzania, rates of facility delivery have remained static for more than 20 years. With an aim to advance research and inform policy changes, this paper builds on a growing body of work that explores dimensions of and responses to disrespectful maternity care and abuse during childbirth in facilities across Morogoro Region, Tanzania.

**Methods:**

This research drew on in-depth interviews with 112 respondents including women who delivered in the preceding 14 months, their male partners, public opinion leaders and community health workers to understand experiences with and responses to abuse during childbirth. All interviews were recorded, transcribed, translated and coded using Atlas.ti. Analysis drew on the principles of Grounded Theory.

**Results:**

When initially describing birth experiences, women portrayed encounters with providers in a neutral or satisfactory light. Upon probing, women recounted events or circumstances that are described as abusive in maternal health literature: feeling ignored or neglected; monetary demands or discriminatory treatment; verbal abuse; and in rare instances physical abuse. Findings were consistent across respondent groups and districts. As a response to abuse, women described acquiescence or non-confrontational strategies: resigning oneself to abuse, returning home, or bypassing certain facilities or providers. Male respondents described more assertive approaches: requesting better care, paying a bribe, lodging a complaint and in one case assaulting a provider.

**Conclusions:**

Many Tanzanian women included in this study experienced unfavorable conditions when delivering in facilities. Providers, women and their families must be made aware of women’s rights to respectful care. Recommendations for further research include investigations of the prevalence and dimensions of disrespectful care and abuse, on mechanisms for women and their families to effectively report and redress such events and on interventions that could mitigate neglect or isolation among delivering women. Respectful care is a critical component to improve maternal health.

## Background

Pregnancy and childbirth continues to place women at risk of significant morbidity and mortality, particularly in sub-Saharan Africa. Globally, in 2010, of 287,000 maternal deaths, 162,000 occurred in sub-Saharan Africa [1]. For every woman who dies of pregnancy-related causes, 20 to 30 others experience acute or chronic morbidity [2, 3]. Efforts to reduce maternal morbidity and mortality emphasize facility-based childbirth and skilled attendance at birth with timely referral for emergency obstetric care if complications occur [4]. This priority is echoed in Millennium Development Goal 5 to improve maternal health, which measures success by tracking the proportion of births conducted with a skilled attendant [5].

Despite decades of efforts to encourage facility births, many women continue to deliver at home. Investigation regarding the barriers that women face in accessing and receiving quality care has long been on the research agenda and emphasized delays particularly related to cost and distance [6, 7]. A more recent emphasis has centered on quality of care and, more specifically, women’s experience of disrespectful care and abuse related directly to provider actions [8–10].

As a concept, disrespect or abuse toward patients in health facilities has proven multidimensional and challenging to define. Similar to concepts such as quality of care or patient satisfaction, the meaning of abuse is subject to variation based on setting, time, birth outcome and personal expectations or opinions. As recently as 10 years ago, nearly no literature addressed the topic [11]; and abuse during childbirth was described as an “emerging problem” [9]. Since then the topic has garnered broader attention with studies in South Africa [12], Ghana [13, 14], Malawi [15], Nicaragua [16], Guatemala [17] and Denmark [18].

In Tanzania, several studies highlight the importance of quality of care during childbirth [19–25], however the experience of abuse, its manifestations and responses to it in non-complicated births has been less explored. An anthropological study by Spangler on embodied inequality – or how social and material status unevenly affects the process of seeking and receiving obstetric care – described how poorer Tanzanian women were more likely to deliver alone or with minimal support, to be scolded, berated or discriminated against, and to be subjected to unpredictable fees [25]. In case studies presented in the study, women paid bribes or moved to the floor during delivery [25].

The relevance of health provider abuse within the spheres of maternal health and human rights is crystallized in the 2011 Universal Rights of Childbearing Women [26], which states:

“Because motherhood is specific to women, issues of gender equity and gender violence are also at the core of maternity care. Thus, the notion of safe motherhood must be expanded beyond the prevention of morbidity or mortality to encompass respect for women’s basic human rights, including respect for women’s autonomy, dignity, feelings, choices, and preferences, including choice of companionship wherever possible.”

Proposed domains of abuse have been highlighted in two seminal articles. D’Oliveira’s work divides violence or abuse in health care into four dimensions: neglect; verbal violence, including rough treatment, threats, scolding, shouting, and intentional humiliation; physical violence, including denial of pain-relief when technically indicated; and sexual violence [9]. Bowser’s review outlines a similar framework that includes: physical abuse, non-consented clinical care, non-confidential care, non-dignified care (including verbal abuse), discrimination of patients, abandonment of care, and detention in facilities [8].

Building on existing frameworks and literature, this study explores how rural Tanzanian women and their male partners describe disrespect and abuse experienced during childbirth in facilities and how they respond to abuse in the short or long-term.

## Methods

### Study setting

In Tanzania, the maternal mortality ratio is 454 deaths for 100,000 live births. One in 38 women have a lifetime risk of death due to maternal causes [27] and for every 1,000 births, 4–5 women die from pregnancy-related causes [28]. Nationwide, 50.2% of births are facility-based and 50.6% of all births are in the presence of a skilled attendant [28]. Since the early 1990s, the national rate of facility-based birth has remained below 52.6% [28, 29]. In rural areas, less than half of births are facility-based (41.9%) and 42.3% of all rural births are in the presence of a skilled attendant [28].

This study was based in 16 villages across 4 districts of Morogoro Region, in eastern Tanzania. Compared to national averages, slightly more women in the region deliver in a facility (58%) and more births are attended by a skilled provider (60.6%) [28]. Throughout the country’s Eastern Zone, which encompasses the region, hospitals and health centers are ill equipped to provide basic or comprehensive emergency obstetric care (EmOC). Basic EmOC is available in 11% of facilities and comprehensive EmOC is available in 10% of facilities [30].

In terms of personnel, facilities in Morogoro Region are understaffed, which reflects national trends. The Region’s density of doctors (0.2), assistant medical officers (0.3) and clinical officers (2.1) per 10,000 people attests to severe human resource limitations [30]. Less than half of all facilities in the Zone (47%) have at least 2 qualified providers assigned to a facility to support basic emergency services 24-hours [30]. Supportive management practices, which are critical for supporting quality care, are also limited. While many facilities in the Eastern Zone receive an external supervisory visit (79%), 34% of facilities provide routine staff training and only 25% of facilities provide “supportive management practices” (an external supervisory visit, routine training and personal supervision) [30].

### Study design

This qualitative, cross-sectional study employed in-depth interviews (IDIs) with women, their male partners, community health workers (CHWs) and community leaders. At eight health centers across four districts, health center staff were asked to identify one village with difficult access to the health center, yet within the center’s catchment area. The data collection team then presented the study to leaders in both the village encompassing the health center and the village described as having difficult access. In Tanzania, the long-standing policy has been for every village to have a village health committee, which appoints two CHWs. Leaders interviewed included religious leaders, as well as members of an elected village board and/or village health committee who identified CHWs. Leaders and CHWs were interviewed irrespective of gender, age, education level, or length of service. Leaders as well as CHWs helped identify women in the village who had delivered in the preceding 14 months. In addition, data collectors canvassed the village and invited eligible mothers and fathers to participate. For a breakdown of respondent groups by distance to facility and district, see Table 1.

Women and their partners were eligible if they had delivered a baby within the preceding 14 months regardless of reports on quality of care, or experiences of disrespectful care. An emphasis was placed on identifying women who had non-complicated, normal deliveries. Women who reported severe vaginal bleeding, eclampsia, obstructed labor, retention of placenta, severe anemia or whose births required vacuum or forceps extraction, or cesarean section were not included with the rationale that such births alter not only careseeking behaviors (often necessitating referrals) but also entail a vastly different subjective sense of the birth experience. For discussion on how a birth experience alters later assessment of quality of care (described as “fulfillment theory”), see Bramadat [31]. All women providing consent were interviewed, until 2–4 women had been interviewed for that site.

**Table 1:** **Respondent groups by distance to facility and district**

	Women*	Male Partners**	CHWs	Community Leaders	Religious Leaders	Total
**Characteristic**						
Near to facility (<3 km)	23	12	12	2	6	55
Far from facility (≥3 km)	26	15	8	3	5	57
**Total**	**49**	**27**	**20**	**5**	**11**	**112**
**District**						
Morogoro Rural	10	2	2	1	1	16
Kilosa	10	11	3	1	2	27
Mvomero	17	8	8	2	4	39
Ulanga	12	6	7	1	4	30
**Total**	**49**	**27**	**20**	**5**	**11**	**112**

*Women who delivered a child within the preceding 14 months.

**Includes any male partner regardless of legal marriage status.

### Data collection

Five Tanzanian research assistants fluent in Swahili with graduate-level training in education, public health, and social sciences were trained for five days to collect the data using instruments, which were pre-tested and revised before starting interviews. Training topics included maternal and newborn health, interview techniques, research ethics and qualitative methods. IDIs were recorded and conducted one-on-one, in a private place of the respondent’s choosing following verbal consent. IDIs focused on experiences related to care seeking during a most-recent pregnancy and birth. At the outset of data collection, the research team did not intend to explicitly investigate experiences of abuse, but rather to explore careseeking for birth in facilities. The abuse theme emerged in the earliest interviews, however, and was probed more explicitly as data collection progressed. A supervisor conducted daily debriefing sessions with data collectors to discuss and triangulate key findings, refine lines of inquiry, and identify saturation of themes. A main product of these debriefings were memos, first generated as a version of meeting notes from debriefings and later amplified by the data collection supervisor to incorporate reflexive notes, contextual information and emerging understandings that could be shared and commented upon by the wider research team. Data collection lasted approximately two months during July and August 2011.

### Data analysis

In-country debriefings with national stakeholders following the close of data collection corroborated and refined the framework for thematic analysis. All interviews were recorded and transcribed into Swahili. An initial phase of open, inductive coding on a selection of rich, diverse and representative transcripts was conducted based in part on Grounded Theory [32]. This resulted in the creation of a codebook that was validated by co-authors. A co-author fluent in Swahili and English applied these broad codes to remaining transcripts using ATLAS.ti [33]. Coded data were then translated from Swahili to English and a second phase of detailed coding was undertaken by a social scientist. During the analysis process, a subset of co-authors discussed codes and themes, and drew comparisons across respondent groups and regions, and by distance to facility. This aided in triangulation of findings and provided texture and nuance to descriptions. Drawing on the principles of Grounded Theory, a literature review followed the completion of coding [32].

The study received ethical approval from the Muhimbili University of Health and Allied Sciences and Johns Hopkins School of Public Health Institutional Review Boards. Names used in this paper are pseudonyms to protect the privacy of interviewees.

## Results

At the outset of interviews, respondents across categories described facilities and providers in a positive light, with several women saying “nilihudumiwa vizuri” (I was attended well). Nearly all women, their partners, community leaders and community health workers (CHWs) living both near and far from facilities refer to providers as “experts” who “possess education”, and who know how to use “real medicine”. Following rapport building, and upon probing for details of the delivery experience, respondents would typically qualify earlier assessments and elaborate on negative aspects of services related to childbirth. In other words, if an interviewer asked a woman if she felt she was mistreated during her delivery, she was likely to say no, but she may later provide a vivid account of a provider shouting at her. Language proved especially critical in terms of probing on this topic. The Swahili word for “to abuse” is “kunyanyaswa”, but no woman said kunyanyaswa when describing her experience. Instead, women described how providers lacked valued qualities such as “kunyenyekea” (to act humbly), “kubembeleza” (to soothe) or “ukarimu” (hospitality). Negative experiences were categorized as ‘abuse’ and ‘disrespect’ in the analysis by the researchers. Findings did not vary by distance to facility.

Presented in Table 2 are types of harsh or abusive treatment outlined by respondent groups and arranged into categories as informed by existing frameworks of Bowser [8] and d’Oliveira [9]: feeling ignored or neglected; monetary demands or discriminatory treatment; verbal abuse; and physical abuse. Examples of resource constraints at the facility-level (including an absence of birth supplies, which was mentioned by all respondent groups), and infrastructure limitations (an absence of electricity or sterilization equipment, emphasized by fathers only) are well documented [30] and will not be elaborated in this paper. Following details on types of abuse, we present responses to abuse as described by women and their partners, categorized on a scale from acquiescent to assertive measures.

**Table 2:** **Types of harsh or abusive behavior preceding, during or after childbirth as defined by mothers, fathers, CHWs and leaders**

Feeling Ignored, Neglected or Mistreated		Mother	Father	CHW	Leader
Family feared delivering alone		X	X	X	
Delivery began or completed in absence of any provider or helper		X	X	X	X
Women felt ignored post delivery (no counseling, no help bathing, walking, removing soiled clothes)		X			
Provider turned family away; told to find a local TBA			X		
Provider routinely absent		X			
Provider routinely ignores women (“they told me I should not interrupt their lunch”)		X	X	X	X
Provider refused to wake for a night delivery		X	X	X	X
Provider had no time to explain a concern		X	X		X
Provider told woman to clean delivery room, mattress and/or table on which a woman delivered		X			
Provider delayed referral until it becomes dangerous for mother or difficult for a family to travel			X		
Provider demonstrated favoritism (toward those who are “connected”)		X	X		X
Provider said “there’s nothing to do” during a complicated delivery (belated referral for c-section)		X			
**Unpredictable Financial Demands/Concerns About Money**		Mother	Father	CHW	Leader
Feeling overcharged (see others pay less or paid less previously); bothered by inconsistent pricing		X	X	X	
Being charged a fine for delivering at home		X			
Being forced to wait longer while those with more money are seen first		X	X		
Being charged for a child’s clinic card		X	X		
Feeling pressured or coerced to pay a bribe (“facility entrance fee” “bed charge” “recognition fee”)			X		X
Being required to pay for medicines bought from a nearby pharmacy or from a nurse (referred to as “going the illegal way” *ukaenda ukapita njia* or “doing business” *kufanya kama biashara*)		X	X	X	X
**Verbal Abuse**		Mother	Father	CHW	Leader
Reports of “being shouted at” or “scolded” for:		X	X	X	X
● Being too tired; “not pushing hard enough”	● Having a TBA as an escort to a facility				
● Having too many children; “ruining” one’s body	● Making a special case of oneself or “requesting too much attention”				
● Arriving too early or too late during labor					
● Taking traditional herbs	● Delivering at home in the past				
● Seeking or heeding advice from a TBA	● Delivering at home and then bringing baby to be registered				
● Wearing old or dirty clothes					
**Physical Abuse**		Mother	Father	CHW	Leader
A nurse refuses to remove a drip because a woman is complaining too much		X			
A nurse slaps a delivering woman		X			
A nurse forces a woman to deliver in a “bad” position (“like kneeling with my head down”)		X

**Table 3:** **Responses to disrespectful care as reported by mothers and fathers**

		Mothers	Fathers
Acquiescent	Do nothing (“I have no choice”)	✓	✓
Measures	Return home	✓	
	Reject facilities in favor of home delivery	✓	✓
	Bypass “bad” facilities for “nice” facilities	✓	✓
	Bypass “bad” nurses within a facility	✓	✓
	Find a TBA in village to assist in facility delivery	✓	✓
	Pay the provider (a bribe, “extra money”, “facility entrance fee”)		✓
	Tell a provider directly to be nicer, to respect patients		✓
Assertive	Report the event to an oversight body (seek reprimand or provider transfer)		✓
Measures	Physically assault a provider		✓

### Types of abuse

The most common negative experience described across respondents entailed feeling ignored or neglected. Verbal abuse was also common, but appeared to be less disconcerting among respondents. Physical abuse was rarely mentioned, was discussed by women only and was identified as insufficiently probed during data analysis. In one case, a woman recalled being forced to deliver in an uncomfortable position. In two other instances, women described fears of being slapped during delivery based on reports from others in their communities. Finally, respondents across categories described monetary demands and discriminatory treatment toward those lacking money, which appeared to upset women and their partners equally. For a comprehensive presentation of types of abuse outlined across respondent groups, see Table 2.

### Feeling ignored or neglected

Several women described fear of arriving at a facility and being ignored or delivering without the assistance of the provider. In instances of night deliveries, some providers were described as being at home on the hospital premises, but unwilling or unable to come out to help.

A woman recalled how a group of providers were in her immediate vicinity but unavailable to her until the moment she yelled that the baby “was coming out”. In this case, a nurse arrived, but not in time to put on gloves.

I was calling ‘Nurse, Nurse!’ she reached there and … the baby came out and she ran to catch her. After catching her she held her and then found gloves to wear before continuing with other services. What I see is that providers should be very close (in proximity) to mothers. A laboring mother can deliver at any time.

- Woman, Kilosa District

A majority of women, but none of their partners, rationalized why over-worked providers were unable to provide ideal care. A woman in Ulanga delivered alone (in the absence of any provider or family member), but rather than feeling frustrated or angry, she sympathized with nurses’ difficult working conditions.

The nurse doesn’t allow anyone to enter inside the room. She is usually alone or maybe with another nurse. I never saw any help (during delivery). You must prepare yourself and just go [Laughing] … you can’t blame anyone. That nurse’s condition is hard… My sister-in-law escorted me but could not be in and could not help me. She could just sit and see me how I’m getting into trouble (Laughing). Beyond that, what could she do? She could only hear me screaming and crying “Aiiii!!?! Mama help me!” It was… impossible.

- Woman, Ulanga District

CHWs described listening to women recount neglect during delivery and how the experience of neglect undermines their ability to encourage facility-based deliveries.

There is one mother who I have spoken with quite often. At the hospital, she says she delivered by herself. She says she called the nurse to come, but the nurse said, “Don’t disturb me so much.” So the mother stayed and delivered by herself. … that mother will not go back to the hospital next time.

- CHW, Mvomero District

I advise women that they should deliver at the hospital because if you deliver at home, a baby can get infections. But women deliver at home anyway. At our hospital, with so few nurses, women don’t want to reach a facility and then start searching for nurses. So a mother decides to just stay home and call the TBA.

- HW, Ulanga District

CHWs and religious leaders expanded on the theme of being ignored and elaborated on versions of verbal abuse. While a CHW used the term “wanaharasiwa”, – forcing an English verb “to harass” into Swahili - this term did not emerge in interviews with women. This CHW described how neglect in facilities reinforces women’s desires to deliver at home.

When the community goes to the center, to go and deliver there, they may find that there is no nurse. The family can go to the nurse’s home and say, “We have brought a laboring woman” but the nurse will delay. She stays in her home until it reaches a very late stage and by the time the nurse comes, that woman has delivered by herself. …Mothers and fathers have complained a lot to us, … They say when they go to the health center they are harassed (wanaharasiwa) …. So then when they are just shouting at the pregnant mother … Women say, ‘It’s better if I just go to the TBA. Even if it’s not safe.’

- CHW, Mvomero District

Families devise solutions to contend with being ignored during delivery – namely shifting oneself from a bed to the floor to prevent a baby from “falling down to the ground”, or sending escorts to find a TBA living near a facility to assist with delivery. No respondent described bringing a relative into the labor room and one woman described that option as a violation of hospital policy.

### Discriminatory treatment, unpredictable financial charges and fear of detention

Women and men described situations where they were expected to bring supplies to facilities for delivery and, less often, situations where they pay a “thank you” to providers following a delivery or pay a fine for home delivery. A few respondents across districts and particularly in more remote areas described how certain women could more easily access supplies or services at facilities. These women had a higher social status, knew someone working within the facility or were somehow, for unknown reasons, favored by providers. Women described how nurses could decide - “upon seeing a woman coming to the facility” - whether they would provide prompt services to her. One woman said she was asked whether she had money, and upon answering ‘no’ she was instructed to sit outside where she watched “the women with money” walk past her to receive services (it is unclear in the transcript if this was an antenatal visit or for childbirth). Another woman described how her sister-in-law was told by providers to pay 15,000 shillings (approximately $9 USD) after a complicated delivery but once at the cashier she was told to pay 40,000 shillings (approximately $25 USD) (it is unclear if this occurred at a public or private facility). That experience invoked confusion and frustration in the woman who feared that she may one day experience a similar situation and be forbidden to leave the facility until she had paid (a practice described by two women). The application of fines and fees was recounted for both maternity and other health facility services.

They are very often saying that medicines are available or not available. When someone tells you they aren’t, it’s her siri (secret). She is the only one who knows. She decides when she sees you coming. … This really upsets us…. The obstacles are like these ones of medicines even if there are no medicines what makes me feel bad is the game.

- Woman, Morogoro Rural District

One religious leader described how young women, first-time mothers and those coming from remote or rural areas are especially prone to discriminatory treatment.

I have myself heard many examples especially for the first mothers who are on their first pregnancy. It is frightening for them to be alone. I hear people say, “If you take her to the hospital, no one will attend her because we are rural, so nurses don’t need to wait on us-- we should be waiting on them. The nurses think it is fine to say to us ‘I feel like sleeping’ or to work however they want to work.”

- Religious leader, Morogoro Rural District

Male partners, more so than women, complained about collusion between providers and pharmacists, and complained about supplies being unavailable at facilities, but available in a provider’s home or at a provider-owned pharmacy. While this was described as inappropriate and unfair, a factor that made it particularly problematic was that men could not be certain how much a syringe or a drip would cost from one day to the next and whether a provider would be compelled to charge a “nice price” or a “high price”. Men lamented their struggle to provide funds to cover delivery costs.

The obstacles I face are so big. I have children and they have a mother. The thing that makes us cry so hard is that there is treatment but without money you can’t get it. It is a big problem, the money.

- Male Partner, Morogoro Rural District

### Verbal abuse

Verbal abuse took the form of criticism levied against women. It entailed outright shouting or harsh remarks. Similar to the preferential treatment domain, verbal abuse was discriminatory in nature. Women who were not following the “rules” or were not presenting themselves as “modern women” were more likely to be berated. While some women reported being scolded for not pushing hard enough, making too many demands during labor or arriving too late or too early for delivery, more common criticisms included critiques of a woman’s economic status (such as wearing old or dirty clothes), critiques of her use of traditional remedies (such as drinking herbal teas and medicines, some of which cause uterine contractions) or her history of home delivery.

A woman was yelled at, during her delivery, for having too many children.

The nurse gets angry. She tells you, ‘You have already delivered many children. This is enough! Look at the others who have delivered only twice or thrice and stopped!’ You will (be in the middle of labor) and hear the nurse saying ‘Come and stop having children!’

- Woman, Ulanga District

Several women were either scolded or witnessed scolding of others for engaging in practices such as visiting a TBA or consuming herbal medicines. Being interviewed about the nature of one’s reliance on traditional ways is part of an admission process at one facility where women reported being first ignored and then harangued until they would “admit” to a practice. Consumption of teas with uterotonic properties is disconcerting for providers (likely due to the possibility of precipitous labor and more difficult management of labor); nevertheless women participants perceived comments about their tea consumption as a criticism of their status or home situation.

When you reach there they have the habit of asking what local medicines have you used or… there is one sister … she arrived they started asking her, ‘Have you ever used local medicines?’ and she replied ‘No.’ But then they just left her there. She tried to follow after them… They went again at her ‘Haven’t you ever drunk local medicines?’. She said, ‘Speaking the truth I drank two cups.’ They said, ‘So you like hurting yourselves, but then you come here you give us trouble.’

- Woman, Mvomero District

Several women interpreted being yelled at as a sign that they were disliked. In this case, a woman felt disliked due to her low economic status.

I don’t know why are they shouting. They just shout at us … they don’t like us. Like with our clothes! …. They give you a bad face. They take a look at you and when your clothes are like this and this they chase you away. Yes, they say, ‘You are supposed to have special clothes for pregnancy!’

- Woman, Mvomero District

Women who delivered at home almost uniformly reported expecting to be yelled at or somehow scolded upon presenting their newborn at a facility. In some cases, they also expected to be charged to receive their baby’s registration card or denied a card altogether. One woman described being treated like a “bad child” for delivering at home.

### Physical abuse

Physical abuse was scarcely mentioned and entailed a fear of abuse rather than enacted violence. One woman described fearing that she would get hit or beaten during labor if she yelled too much or “talked back” to a provider. She reported witnessing this behavior among others, but did not experience it herself. Another woman described being told she had to deliver while lying down with her knees pulled “up”, which she found uncomfortable and frightening. One woman described a nurse refusing to remove her drip because she had made a “special case” of herself. Similar to other dimensions of abuse, in the instance of a woman wanting to deliver in a standing position, she was berated for not adjusting her preferences to a “modern” mold. Another woman who was scolded for requesting that her drip be removed, felt certain that “if I was staying with influential people in a place near the facility” the nurses would not have felt emboldened to deny services.

### Responses to abuse

Reponses to abuse stretched across a continuum from acquiescent to assertive measures (see Table 3). Women were more likely than men to describe how they empathized with over-worked providers. Several women described how they watched exasperated nurses rush from ward to ward.

I am not angry… Because you can see that one nurse, she is at the parent’s ward, then at the children’s ward, then at the men’s ward. She may be giving injections from 11 in the morning to 11 at night…. (from) the labor ward, you can see that the nurse has more (people needing) injections waiting for her.

- Woman, Ulanga District

Acquiescent or non-confrontational measures to address abuse during childbirth included: resigning oneself to the experience, returning home, rejecting facilities altogether, or bypassing ‘bad’ facilities or ‘bad’ providers. Among these measures, the preferred option described by women and men was to reject facilities in favor of home birth. Men and women alike described doing nothing or circumventing bad facilities.

Assertive or confrontational measures to address abuse during childbirth included: finding a TBA to assist in a facility delivery, paying a bribe, confronting a provider, reporting an event to an oversight committee or physically assaulting a provider. While women living in remote areas described finding a TBA to assist in a delivery, only men described the remaining active measures to address abuse.

### Resigning oneself to the experience

When probed on reasons for not confronting or addressing abuse, respondents reported fear of retaliation during later visits coupled with uncertainty about what precisely to do to effectively address abuse. One male partner described an inability to complain.

You cannot complain, you need to say thank you. Because they give us drugs, so we can’t complain. And we don’t know who would be accountable to rescue us.

- Male partner, Ulanga District

A fear of repercussions, in particular future denial of care or services were particularly powerful forces working against women’s desires to speak out against abuse.

There is no place you can go, you must keep quiet. … They can hurt you… The routine is set. …. I’m afraid that if I say anything to anyone, I could get reported or not get treatments.

- Woman, Mvomero District

Women also feared that complaints to higher levels of government may lead to facility closures, which would further undercut their access to care.

Some people are saying that if we find the situation is like this, we should make a call to our councillor and go to our regional offices and tell them we are oppressed. But we see a concern if we do this. … what if they close our hospital?

- Woman, Morogoro Rural District

### Delivering at home or bypassing

TBAs were described as having a calming presence during a birth or “removing the fear” of giving birth in spite of what several respondents described as TBAs’ “lack of real medicine”. Going to a TBA’s home or delivering in one’s own home in the presence of a TBA was mentioned by women and their male partners – regardless of distance to a facility – as a means to avoid unpleasant experiences at facilities.

When the TBA is there you can’t be afraid.

- Woman, Mvomero District

In terms of bypassing within a facility, women described a need to be careful in how they frame a preference for a particular provider over another. Some women said that if they see a certain provider offering services, they return home. Others try to avoid eye contact when a disliked provider calls their name. Nevertheless, this mother said that while she has a distinct preference for one provider, she is not able to avoid the provider’s disliked colleague.

It’s better you get attended by a certain person. But you can’t reach there and say, ‘I don’t like you. I would like you instead to attend me.’ That I can’t say. But I know it’s better when I go to this other person. She is much more polite.

- Woman, Morogoro Rural District

### Payments, lodging a complaint, assaulting a provider

Only men described paying bribes or fees. A few men considered bribes a necessary process in order to “be seen” by providers.

If you don’t have money, they look at you as if you are not there. They leave you like that. So we prepare. As you know it’s just about money so we prepare and then go.

- Male partner, Ulanga District

While reporting abusive behavior was seen by women and men as an “official” path, it was also deemed largely impractical (given a potential backlash) or ineffective (as it would likely remain ignored). Nonetheless, in one village, community leaders described how a problematic provider was transferred from her post following a series of complaints lodged on behalf of the community via a village health committee.

The most extreme response to abuse was described by one male partner, who witnessed a man attacking a provider for insisting on a bribe before treating his laboring wife.

That doctor was beaten by one man. He said to the doctor, “I do not have that money. But she needs those services”. The doctor said, “Go get some money.” He went home and found some money. Then he gave the doctor the money. When that doctor took his money, the man just … hit him. He beat him hard. That man said to the doctor, “It is your job to be our doctor. Not to take bribes.” And then he just started beating. On the neck. On the face. He was beating him. … This was a beating from our community. People are tired of this. Investigators came after the beating and the doctor was transferred. After that beating the services got better… that doctor left and a lot of the problems with bribing left, too.

- Male partner, Morogoro Rural District

## Discussion

This study explored, in detail, across a wide range of respondents, how women and their families experience and respond to abuse during childbirth in rural Tanzanian health facilities. We found that all respondent groups regardless of gender, distance to facility or district reported negative experiences that align with existing classifications of abuse or disrespectful care [8, 9, 26]. The domains of abuse described in our paper align with several domains outlined by Bowser including: non-dignified care (including verbal abuse), discrimination, abandonment of care and detention in facilities. Bowser’s categories of physical abuse, non-consented care, non-confidential care and outright physical violence did not emerge as strongly in this study. Our study also found that abuse can be ambiguous, difficult for respondents to articulate and subject to “personal yardsticks” or pre-conceived expectations [31]. Many respondents in this study report satisfaction with facility-based childbirth while at the same time describe being discriminated against, ignored or verbally abused. This paradoxical finding is echoed in quality of care literature, which highlights that satisfaction (as a feeling or affect) does not necessarily align with perception (a cognition), and that patients whose expectations barely extend beyond a provider’s physical attendance at birth can often assess a low quality experience as satisfactory [31].

This study draws together and is corroborated by several studies in Tanzania that describe poor quality of care, and to a lesser extent abuse, as factors guiding delivery preference. Kruk’s discrete choice experiment found that a provider’s attitude and the availability of drugs were the most important characteristics influencing choice of a facility delivery and that improving these characteristics would lead to a 43-88% increase in facility delivery [20]. Mrisho’s research found that staff attitudes including abusive language, denial of service, and an absence of compassion represent one among many barriers to facility-based care, which drives women to deliver at home [22]. Several studies, in particular the work of Spangler, have highlighted how women recognize and internalize feelings of discrimination because they are under-dressed, rural, cannot afford a bribe or lack political or social influence [21, 25, 34]. Reports of being charged fees (also called “under-the-table”, or “asante” (thank you) charges) even in facilities that are officially exempt from payment, has also been detailed [25, 34, 35]. Being charged fines for home deliveries has been instituted as an unofficial practice in some communities as a means to compel facility delivery, however no respondent was aware of such by-laws and instead view fines as a form of discrimination. Most recently, Mselle’s qualitative study in Dar es Salaam and Dodoma regions found that poor quality care and poor working environments contributed to “bad birth experiences” which “undermine the reputation of the health care system, lower community expectations of facility birth, and sustain high rates of home deliveries” [23]. While each study has described an attribute or component of disrespect, Mselle was among the first to explicitly examine abuse and its dimensions in this context. That study, however, drew on data from women with negative birth outcomes (obstetric fistula). Negative outcomes portend over-reporting of negative experiences (also termed “fulfillment theory” [31]), which highlights a need for data that draws on experiences in non-complicated, healthy, vaginal deliveries.

Responses to abuse highlighted in this study range from acquiescent to assertive measures. Women tend to report preferring non-confrontational approaches and expressed empathy toward over-worked providers. Men may not have reported such empathy as they spend relatively little time in health facilities. Our findings correspond with other articles that emphasize that a woman’s or family’s “delay” in seeking care in facilities is not always an oversight borne of lack of knowledge or education, but an active decision made by her and in cooperation with others in her community based on previous experience and an effort to take a course of action deemed to be in the best interest of her and her baby [36].

**Figure 1:**
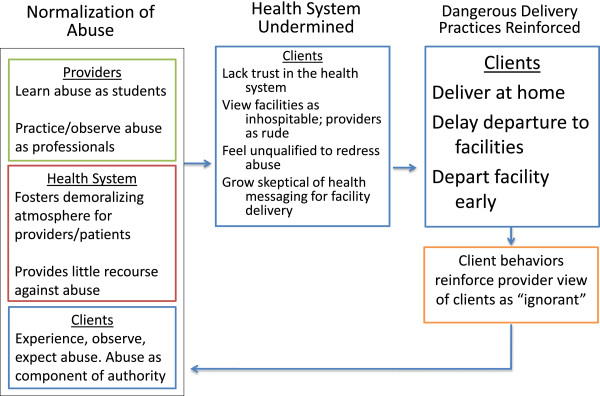
**Pathways from disrespectful care to dangerous delivery practices.**

Our findings, when placed within the context of existing literature, illustrate a cyclical nature of abuse (see Figure 1) - how abuse becomes normalized and expected, how its existence undermines patients’ views of facilities and providers and how these negative attitudes weaken efforts to encourage careseeking in facilities for birth. As described in leading abuse literature and frameworks [9, 11] providers may engage in disrespectful care because they learned or observed this during pre-service training, because they are faced with severe human resource or supply limitations (and are contending with resultant poor motivation) or because they have “internalised dominant cultural values and beliefs regarding gender and gender-based violence” [37]. Clients, including many respondents in this study, then perceive facilities as harsh environments and either reject them altogether or attempt to minimize engagement with the formal health system by delivering at home, departing late for facilities, or leaving facilities very early after delivery.

Using Figure 1 as a guide, we prioritize interventions that address the normalized nature of abuse. Providers, women and their families must be made aware of women’s rights to respectful care. Existing documents including a Code Of Professional Conduct For Nurses And Midwives In Tanzania outline principles of dignity, respect, consent, professionalism, accountability and honesty [38]. For providers, we view participatory trainings (that ideally draw from carefully crafted professional codes) as opportunities to reflect on biases and to work together to resolve existing problems. Trainings must be supported by management and other levels of the health system in order to be effective in enacting a zero tolerance policy toward abuse [39]. For women and their husbands, we found that limitations related to reporting concerns in a private, safe and effective manner fostered a sense that providers were beyond redress [40–42]. Research on how to improve respectful care, responsiveness and accountability is warranted.

At the facility level, efforts to improve the working environment of providers must be made in terms of general infrastructure improvements, addressing human resource shortages and remedying deficiencies in supervision and skills training [43]. Providers often want to provide quality care, but lack the resources to make this possible [43]. A key finding from this study revealed that women feel neglected or ignored during birth. Facilities need to revisit the inclusion of family members or birth companions during labor or delivery. As labor wards are open, one reason companions are currently excluded relates to privacy considerations for other laboring women. However, Shimpuku’s qualitative study concluded that in the midst of crowded facilities staffed by overworked nurses, families play a critical role in advocating for “invisible” laboring women [24]. Respondents in this study discussed how escorts and companions assist women and advocate for them as providers are often absent. If it is possible for families to be with women, while maintaining privacy and respect for others, we view this as a critical opportunity to minimize women’s fear and enhance their comfort. Promoting birth companions is not without challenges and further studies on how to do this in a manner that is feasible, acceptable and appropriate are necessary [44].

### Limitations and opportunities for future research

Due to the nature of qualitative research, abuse was not evenly probed in each interview. This limited our ability to systematically assess the relative importance or value that husband-wife pairs place on a particular aspect of abuse. Second, this study relied on reported experiences rather than direct observation. Third, this study captured insights from women who, in some cases, delivered several months earlier and may therefore have a recall bias.

We recognize several opportunities for future research. First, this study did not reach saturation on characteristics of women that could have informed analysis including: age, parity, socioeconomic status, relationship and gender of facility escort, and how a woman would characterize her (or her family’s) relationship to or previous experience within a facility. Second, we did not interview providers, who could have shared a critical understanding of whether, how and why they engaged in disrespectful care or abuse. Providers in this context experience severe professional and personal constraints themselves, which can affect whether and how they interact with patients [45]. Lacking adequate personnel and equipment, and working without payment in facilities that lack basic necessities, providers may pass their frustrations on to their patients [9]. Third, we did not purposively identify and interview facility escorts (mothers, sisters, or in-laws who accompany women to facilities), who could have provided more extensive information about the periods preceding and during delivery, as several women had difficulty recalling the time period before, during and after delivery. Fourth, looking ahead, we recommend further research that can better capture nuances and terminology related to quality of care and respectful maternity care and following this we recommend incorporation of questions related to quality of care into population-level surveys such as the Tanzania Demographic and Health Survey, which aims to assess barriers related to accessing health care for delivery.

## Conclusions

Tanzania is not on the path to realizing MDG 5 [46]. Tanzania’s health care system is facing a critical dilemma as it tries to balance demands to increase facility deliveries, while also contending with severe staffing shortages and infrastructure constraints. The Government must address constraints in facilities in order to improve the environment for providers delivering services and women receiving care. We recommend implementation research on health system strengthening strategies that bolster the provision of respectful quality care by supporting synergies across provider training and supportive supervision, problem solving for health system constraints, community and client awareness-building regarding patient rights and venues to seek redress, and the inclusion of escorts during labor and delivery could all be considered as opportunities to build trust in facilities. At present, many Tanzanian women experience highly unfavorable births in facilities, which may play a critical role in the stagnation of facility-based births in recent decades, particularly in rural areas. Respectful care is a vital component to addressing Millennium Development Goals to improve maternal health.
